# 

**Published:** 1856-04-01

**Authors:** 


### CONTENTS.

PAGE

PSYCHOLOGICAL QUARTERLY RETROSPECT . . . . . . . . . . xxv—lxii

PART I.—ORIGINAL COMMUNICATIONS.

1. Medical Jurisprudence of Insanity:—

   Part I.—On Lucid Intervals . . . . . . . . . . . . . . 144

2. Ethnological Psychology . . . . . . . . . . . . . . . . . 171

3. Notes of a Visit to the Public Lunatic Asylums of Scotland . . . 183

4. Autobiography of the Insane . . . . . . . . . . . . . . 202

5. Physiological Psychology . . . . . . . . . . . . . . . . 226

6. On Moral and Criminal Epidemics . . . . . . . . . . . . 240

7. On some Unrecognised Forms of Mental Disorder . . . . . . . . 282

PART II.—FOREIGN PSYCHOLOGICAL LITERATURE.

1. On the Detection of Doubtful Insanity . . . . . . . . . . . 291
2. Suicide amongst Children . . . . . . . . . . . . . . . . 296
3. The Theory of Automatism, with the Manuscript of a Monomaniac . 299
4. Practical Observations on the Domain of Psychiatry, with a Retrospect of the last Thirty Years . . . . . . . . . . . . 301
5. On the Causes of Insanity . . . . . . . . . . . . . . . . 305

---

On Criminal Responsibility . . . . . . . . . . . . . . . . 305

The Parish of St. Pancras and its Lunatic Poor . . . . . . . . . 310

